# Heterologous expression of antigenic peptides in *Bacillus subtilis* biofilms

**DOI:** 10.1186/s12934-016-0532-5

**Published:** 2016-08-11

**Authors:** Cédric M. Vogt, Elisabeth M. Schraner, Claudio Aguilar, Catherine Eichwald

**Affiliations:** 1 Institute of Virology, University of Zurich, Winterthurerstrasse 266a, 8057 Zurich, Switzerland; 2 rqmicro Ltd, ETH, Otto-Stern-Weg 7, 8093 Zurich, Switzerland; 3 Institute of Anatomy, University of Zurich, Zurich, Switzerland

**Keywords:** *Bacillus subtilis*, TasA, Biofilm, Endospores, Heterologous protein, mCherry, *E. granulosus*, Tropomyosin, Paramyosin, Antigen

## Abstract

**Background:**

Numerous strategies have been developed for the display of heterologous proteins in the surface of live bacterial carriers, which can be used as vaccines, immune-modulators, cancer therapy or bioremediation. Bacterial biofilms have emerged as an interesting approach for the expression of proteins of interest. *Bacillus* s*ubtilis* is a well-described, endospore-forming organism that is able to form biofilms and also used as a probiotic, thus making it a suitable candidate for the display of heterologous proteins within the biofilm. Here, we describe the use of TasA, an important structural component of the biofilms formed by *B. subtilis,* as a genetic tool for the display of heterologous proteins.

**Results:**

We first engineered the fusion protein TasA-mCherry and showed that was widely deployed within the *B. subtilis* biofilms. A significant enhancement of the expression of TasA-mCherry within the biofilm was obtained when depleting both *tasA* and *sinR* genes. We subsequently engineered fusion proteins of TasA to antigenic peptides of the *E. granulosus* parasite, paramyosin and tropomyosin. Our results show that the antigens were well expressed within the biofilm as denoted by macrostructure complementation and by the detection of the fusion protein in both immunoblot and immunohistochemistry. In addition, we show that the recombinant endospores of *B. subtilis* preserve their biophysical and morphological properties.

**Conclusions:**

In this work we provide strong evidence pointing that TasA is a suitable candidate for the display of heterologous peptides, such as antigens, cytokines, enzymes or antibodies, in the *B. subtilis* biofilms. Finally, our data portray that the recombinant endospores preserve their morphological and biophysical properties and could be an excellent tool to facilitate the transport and the administration.

**Electronic supplementary material:**

The online version of this article (doi:10.1186/s12934-016-0532-5) contains supplementary material, which is available to authorized users.

## Background

Live bacteria carriers have been used for several years to display heterologous proteins in their surface such as antigens, cytokines, enzymes or small-immune proteins for different purposes including vaccines, immunomodulators, cancer therapy or bioremediation. Several strategies have been described for the expression of heterologous proteins in the surface of bacterial carriers, as are: (i) the fusion of a target protein to surface proteins from gram-positive or gram-negative bacteria [1–4]; (ii) expression via the autotransporter adhesion involved in diffuse adherence [5]; (iii) using the LPTXG anchoring motif to present antigens in the surface of gram-positive bacteria [6] or (iv) via outer membrane vesicles as is for gram-negative bacteria [7]. Besides the method used to display a heterologous protein, it is highly desired that the expression of this protein be enhanced at the best of its capacities. For example, inducible promoters have been implemented that can drive the expression of a heterologous protein and, at the same time, amplify the expression plasmid carrying the recombinant gene. Such promoters need to be activated at the micro-environmental conditions encountered by the live bacteria carriers. Conversely, if the heterologous expression needs to be halted, like for in vivo antigen expression, the use of common antibiotics or the introduction of inducible suicide genes can be used [8, 9].

Biofilms are communities of surface-associated microorganisms encased in a self-produced extracellular matrix that commonly comprises lipids, proteins that frequently exhibit amyloid-like properties, eDNA and exopolysaccharides. This matrix fulfills a variety of functions for the community, from providing structural rigidity and protection from the external environment to controlling gene regulation and nutrient adsorption. Thus, the gain of knowledge about the bacterial communities is making the biofilm an attractive tool for the display heterologous proteins in their surface. Even if several uncharacterized proteins are present in the *Bacillus subtilis* biofilm matrix, two important structural proteins have been described so far: TasA (*t*ranslocation-dependent *a*ntimicrobial *s*pore component) and BslA (*b*iofilm *s*urface *l*ayer protein) [10]. TasA, besides being a major component of the extracellular matrix biofilms, forms amyloid-like fibers that have two proposed roles: (i) detoxification of potential aggregates of this protein in the cytoplasm and (ii) to form the protein-scaffold that supports the assembly of the extracellular matrix [11]. The *tapA*-*sipW*-*tasA* operon is necessary for the formation of the amyloid fibers, where TasA stands as main component [12]. Unlike the wild type, mutants in *tasA* are unable to form structured and complex biofilms, developing only in featureless and flat colonies when grown under biofilm-inducing conditions [13]. TapA (**T**asA **a**nchoring and assembly **p**rotein) is an accessory protein that promotes the efficient polymerization of TasA at the cell envelope, contributing to the organization of the growing fibers and acting as connector of the fibers to cell envelope [14]. In addition, SipW is a signal peptidase required for TapA and TasA processing and secretion [13, 15]. Upon starvation conditions, *B. subtilis* has the capacity to form endospores, a dormant form of life with the potential to disperse until environmental conditions that are propitious for germination are encountered. It is important to denote that *B. subtilis* spores have been proposed as probiotics for animal consumption [16, 17] and in humans for diarrhea treatment and the eradication of *H. pylori* [18]. Interestingly, it has been observed that in a *C. elegans* gut model, *B. subtilis* can interact with host pathways in the nitric oxide synthesis, leading to extension of worm lifespan [19, 20]. In the case of recombinant *B. subtilis* spores, they have been proposed as carrier of heterologous proteins by direct attachment to surface coat proteins (CotB, CotC, CotG, OxdD, SpsC, CotA and CotZ) for diverse applications ranging from oral vaccines vehicles to bioremediation tools, and including biocatalysts, as well as the generation and screening of mutagenesis libraries. In addition, a non-recombinant approach has been recently developed to adsorb antigens and enzymes on the spore surface [21]. Thereby, the well-known biology and genetics of *B. subtilis* plus its capacity to form both biofilms and endospores as well as acting as probiotic, make of this gram-positive bacteria a suitable candidate for the display of heterologous proteins.

Here, we propose an efficient method for the display of heterologous proteins in the surface of *B. subtilis* biofilms. In our strategy, we show that the red fluorescent protein mCherry and also antigenic peptides from *E. granulosus* parasite, EgTrp and EgA31, can be efficiently exposed in the biofilm matrix by direct fusion to the C-terminus of TasA. We also demonstrate that the spores obtained from our recombinant *B. subtilis* strains are biophysically and morphologically identical to wild type spores.

## Results and discussion

**Fig. 1:**
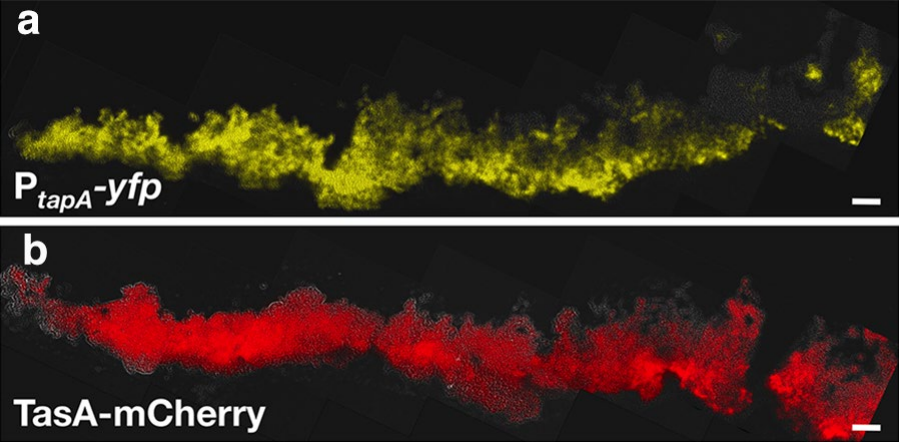
Spatial distribution of TasA within the biofilm. Vertical thin sections of biofilms harboring a *yfp* reporter fusion to P_*tapA*_ (**a**) or a TasA-mCherry fusion protein (**b**). Biofilms were frozen at 72 h of development prior to cryosectioning and fixation. Images represent partial colonies, the edge is shown at the *left* and the agar surface is at the *bottom*. Transmitted light images were overlaid with fluorescence images that were false-colored for each reporter. *Bar* 50 µm

**Fig. 2:**
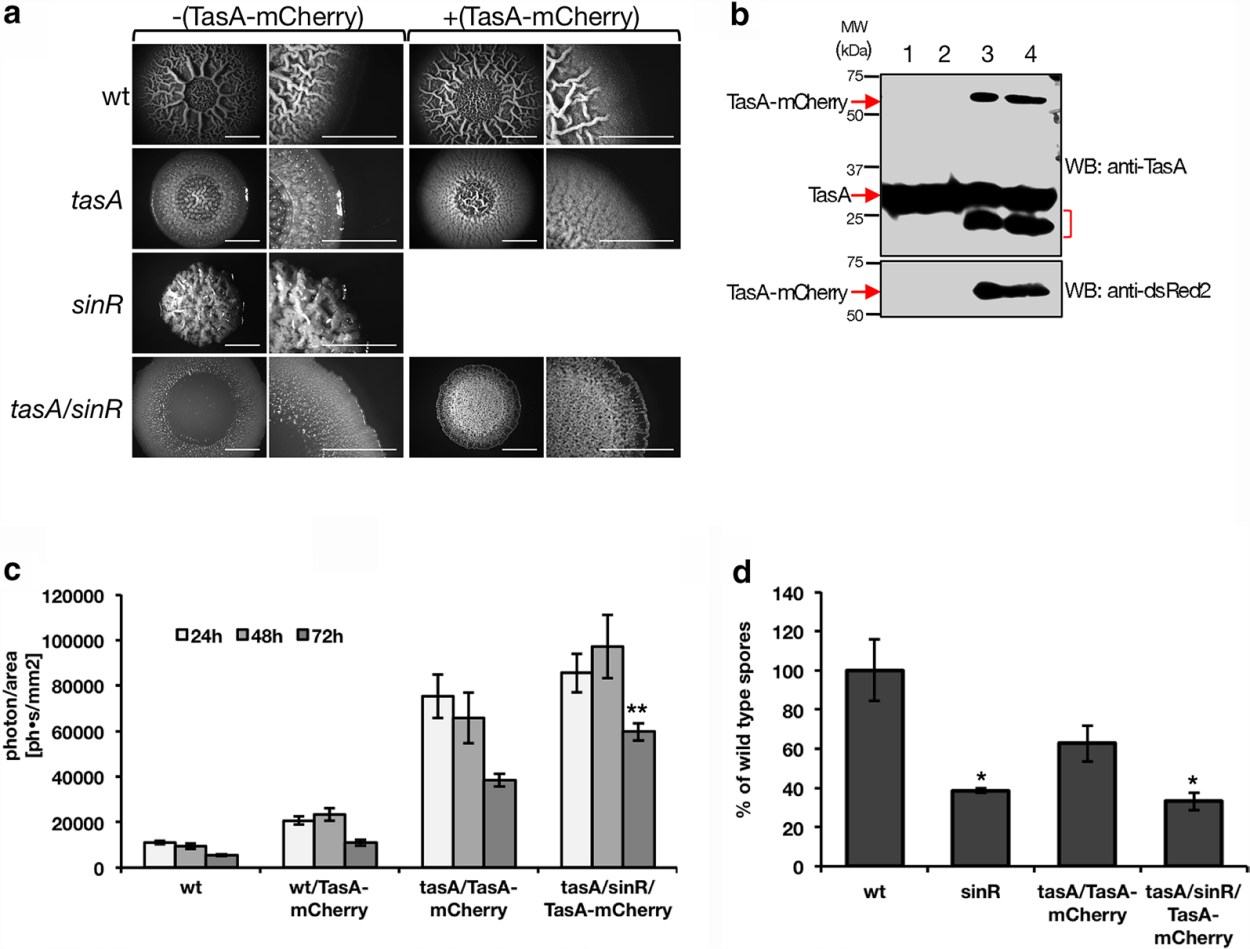
**a** *Top view* of biofilm architecture from wild type, *tasA* and *tasA/sinR* strains with negative (*left panels*) and positive expression of TasA-mCherry (*right panels*) after 72 h of incubation. A magnification of the edges of the colony is presented at the *right* of each the whole colony top view picture. *Scale bar* is 1 cm. **b** Immunoblotting of biofilm extracts from wild type (*lane 1*), *sinR* (*lane 2*), *tasA*::TasA-mcherry (*lane 3*) and *tasA*/*sinR*::TasA-mCherry (*lane 4*) strains; TasA and TasA-mCherry were detected using specific anti-TasA (*upper panel*) and anti-dsRed2 (*lower panel*) antibodies. The *red arrows* indicate the position of TasA and TasA-mCherry. The *red bracket* indicates an anti-TasA reactive band with a lower molecular weight of TasA, presumably TasA degradation. The molecular weights (kDa) of the proteins are indicated. **c** Red fluorescence quantification of 24, 48 and 72 h biofilms. Data is presented as the mean ± SEM of four independent experiments. *Asterisks* denote significant differences in TasA-mCherry red fluorescence between *tasA* and *tasA*/*sinR* strains expressing TasA-mCherry (*t* test, **p < 0.001, n ≥ 4). **d** Viable spore counts comparing *sinR*, *tasA*::TasA-mCherry and *tasA*/*sinR*::TasA-mCherry to wild type percent of spores in biofilms. *Error bars* indicate SEM (t-test, *p < 0.05, n = 4)

*Bacillus subtilis* NCIB3610 is a non-domesticated strain employed in many studies of bacterial development for its ability to form architecturally complex structures called biofilms. This strain is very closely related to the widely used (domesticated, non-biofilm forming) laboratory strain *B. subtilis* 168. Thus far, mutations in several genes have been identified in *B. subtilis* 168 which have been shown to contribute to biofilm development in *B. subtilis* NCIB3610 [22–24]. In this study, we aimed at the expression of heterologous peptides in *B. subtilis* NCIB3610, taking advantage of its remarkable ability to form biofilms. The formation of biofilms is partially dependent on the activation of the *tapA*-*sipW*-*tasA* operon [10, 25]. This activation can be readily monitored within the biofilm by using a transcriptional fusion of the promoter of this operon (P_*tapA*_-yfp) to a reporter gene like the fluorescent protein *yfp* [26]. Our results were in perfect agreement with previous observations regarding the expression of this operon in the biofilm (Fig. 1a). Since the expression of the *tapA* operon leads to the production of TasA, one of the proteins crucial for biofilm development in *B. subtilis* [13, 26], we next asked if the expression of the operon could be correlated to the presence of TasA in the biofilm. To do this, we engineered a protein fusion between TasA and the red fluorescent protein, mCherry. As depicted in Fig. 1b, TasA-mCherry displays a massive distribution within the biofilm matrix at 72 h of biofilm development, in agreement with its suggested role in providing structural support within the biofilm matrix [12, 13]. Based on the abundant and homogeneous distribution of TasA-mCherry in the biofilm, we hypothesized that TasA may be an excellent candidate for exposing heterologous proteins, such as antigenic peptides, enzymes, immunodulators or even small-immune molecules. In order to optimize the best conditions of expression of a TasA fusion protein, we first monitored the abundance of TasA-mCherry, engineered as a *tapA*-*sipW*-*tasA*-*mCherry* operon in different genetic backgrounds. We compared the expression of this operon in both the *B. subtilistasA* mutant and in a double mutant for *tasA* and *sinR* (*tasA*/*sinR*) strains, to the expression of the operon in wild type *B. subtilis*. The mutant *tasA* has been already described to only form featureless colonies [26, 27]. Thus, when expressing TasA-mCherry in the absence of endogenous TasA, we can observe a recovery of the biofilm macrostructure architecture (Fig. 2a, middle row). On the other hand, SinR is a repressor of the *eps* and *tapA*-*sipw*-*tasA* operons whose function is antagonized by SinI [13, 28]. In addition, SinR is only active in a biofilm in cell subpopulations that express *tasA*-*sipW*-*tapA* and *eps* operons [26, 29]. Thus, a mutant in *sinR* develops biofilms with an exacerbated architecture rich in wrinkles and able to retain water, partially due to an overexpression of the *eps* and *tapA*-*sipw*-*tasA* operons (Fig. 2a, left panel). On contrast, the double mutant *tasA/sinR* develops in a biofilm with a less complex architecture, as depicted in Fig. 2a (lower row), with an overall flat architecture, smooth edges and no evident macrostructures. These results are in further agreement with the role of TasA as a structural matrix protein. We then expressed the *tapA*-*sipW*-*tasA*-*mCherry* operon in both the *tasA* and the *tasA/sinR* strains. Our results indicate that the expression of the fusion protein TasA-mCherry was enough to at least partially reconstitute the biofilm architecture in both *tasA* and *tasA/sinR* mutant strains (Fig. 2a, middle and lower rows). An immunoblotting of biofilm extracts expressing TasA-mCherry in *tasA* and *tasA/sinR* strains (Fig. 2b, lanes 3 and 4) exhibit a band with an apparent molecular weight of 50 kDa that was detected with either anti-TasA or anti-dsRed2 and that corresponds to the predicted molecular weight for the fusion TasA-mCherry. Similar results were obtained for the reconstitution of biofilms as well as for the expression of TasA-mCherry in pellicles (Additional file 1: Figure S1a, c). All together, these results indicate that TasA can be in fact expressed as a fusion protein within the biofilm and that, importantly, the “cargo” did not significantly alter the functionality of TasA fused to it, in this case mCherry. Based on these observations we were encouraged to further characterize different physiological and anatomical aspects of *B. subtilis* when expressing TasA as a fusion protein.

We next identified the best strain for the heterologous expression of a peptide fused to the C-terminus of TasA. For this purpose, we quantified the red fluorescence emitted by the TasA-mCherry protein, monitoring the development of biofilms formed by either the wild type, *tasA* and *tasA/sinR* strains expressing the *tapA*-*sipW*-*tasA*-*mCherry* operon at 24, 48 and 72 h (Fig. 2c). Our data showed a significant increase in fluorescence in biofilms formed by the *tasA* or the *tasA/sinR* strains over the wild type strain when expressing *tasA*-*sipW*-*tasA*-*mCherry* operon at all measured times of biofilm development. We also observed that, in contrast to *tasA* strain, the *tasA/sinR* strain displays a persistent and strongest fluorescence signal up to 72 h. Based on these results, we conclude that the *B. subtilis tasA/sinR* strain allows for the best conditions for expression of a heterologous protein.

Upon harsh conditions *B. subtilis* has the ability to form endospores, which are highly resistant to environmental offences [30] and are able to provide protection to the encapsidated bacterial genome until the propitious conditions for germination are encountered. Since the heterologous peptide is encoded in the genome of *B. subtilis*, it was then reasoned that the endospores could be used as delivery agents of the fusion protein. For example, if orally administrated, the endospores can bypass the acidic stomach barrier and may later germinate in the gut, only then expressing the fusion protein of interest. This strategy would at least partially circumvent the problems encountered when spore-coat proteins carried an antigenic peptide, which could be severely damage the integrity of the peptide [31]. By using high-resolution electron microscopy, we compared the ultrastructure of the endospores formed by the wild type, *tasA*, *sinR* and *tasA/sinR* strains (Additional file 2: Fig. S2). We could not detect significant structural differences in these endospores, indicating that the lack of TasA or SinR did not affected the integrity of either the spore coat or the peptidoglycan cortex. Also, we then compared the sporulation efficiency in biofilms formed by the different strains expressing TasA-Cherry (Fig. 2d). As expected, wild type and *tasA*/TasA-mCherry strains show comparable sporulation abilities [26]. On contrast, we observed a reduction in the sporulation ability of the *sinR* and *tasA/sinR*/TasA-mCherry strains. These data were in agreement with previous observations from Veening et al. [32], explained by a premature activation of the unidirectional gene cascade of feed-forward loops governing spore formation [33]. This reduction in the spores number observed between wild type and *tasA/sinR*/TasA-mCherry, however, was not detected when liquid cultures [34] were used instead of biofilms for spore preparation (Additional file 3: Fig. S3). Our results indicate that the best genetic background for the expression of heterologous peptides in biofilm of *B. subtilis*, when in frame with the 3′ end of the *tapA*-*sipW*-*tasA* operon, is the *tasA/sinR* strain.

**Fig. 3:**
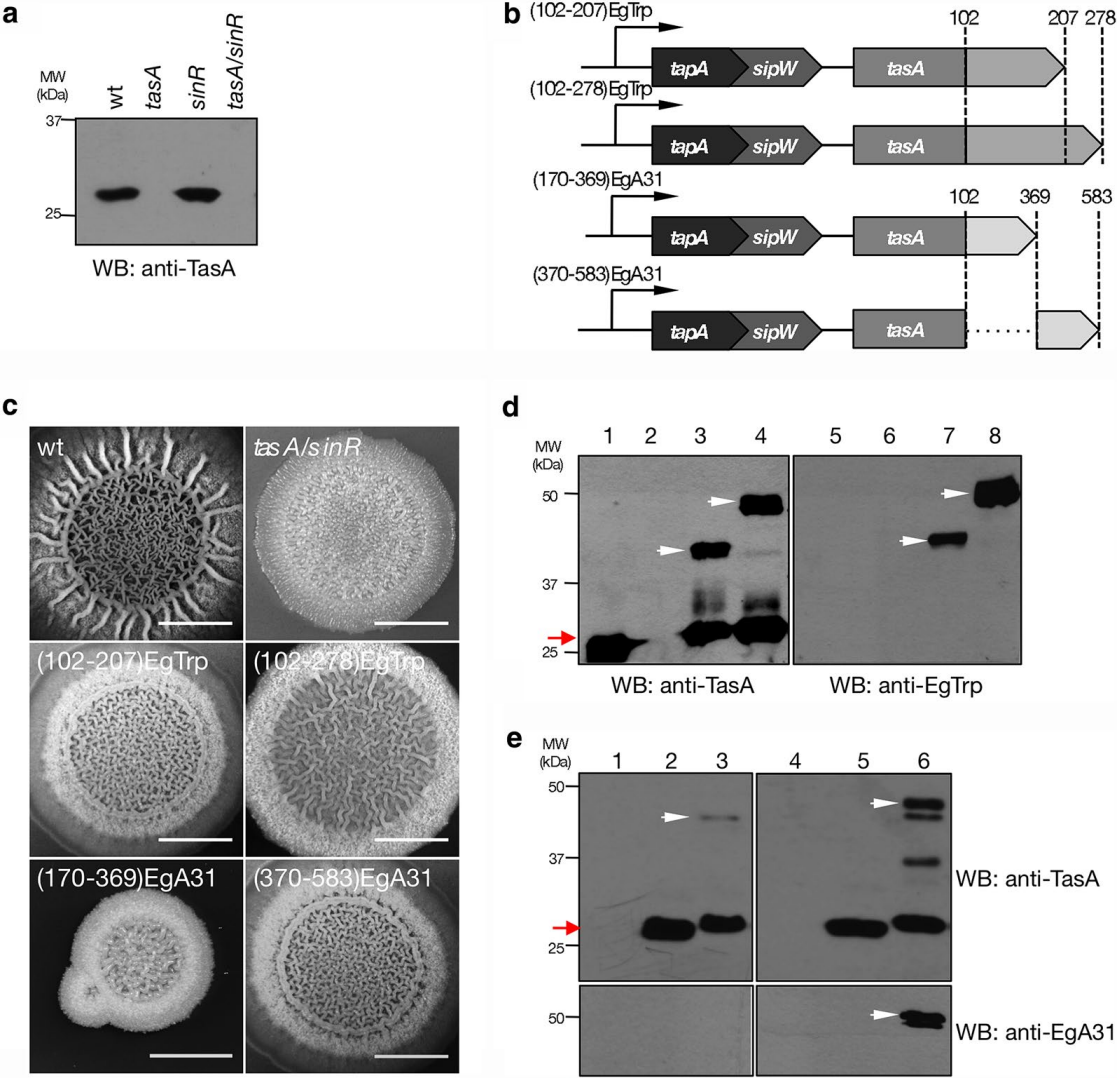
**a** Immunoblotting of biofilm extract at 72 h for wild type, *tasA*, *sinR* and *tasA*/*sinR* (single antibiotic selection) strain for the detection of TasA using an anti-TasA antibody. **b** Schematic representation of *tapA* operon carrying *E. granulosus* antigenic peptides, EgTrp and EgA31, fused in frame at the 3′ end of *tasA*. *tapA*, anchoring and assembly protein; *sipW*, signal peptidase and *tasA*, major protein matrix. The amino acid region corresponding to each antigenic peptide is indicated. For simplicity of the figures, TasA-(102-207)EgTrp, TasA-(102-278)EgTrp, TasA-(170-369)EgA31 and TasA-(370-583)EgA31 are named as (102-207)EgTrp, (102-278)EgTrp, (170-369)EgA31 and (370-583)EgA31, respectively. Diagram not to scale. **c** *Top view* of biofilm architecture from wild type, *tasA*/*sinR*, TasA-(102-207)EgTrp, TasA-(102-278)EgTrp, TasA-(170-369)EgA31 and TasA-(370-583)EgA31 strains at 72 h. *Scale bar* is 1 cm. **d** Immunoblotting of biofilm extract for wild type (*lanes 1 and 5*), *tasA*/*sinR* (*lanes 2 and 6*), TasA-(102-207) EgTrp (*lanes 3 and 7*) and TasA-(102-279)EgTrp (*lanes 4 and 8*) strains detected with anti-TasA (*left panel*) and anti-EgTrp (*right panel*) antibodies. The *white arrowhead* indicates the position of the TasA-EgTrp antigenic peptides and the *red arrow* indicates the position of TasA. **e** Immunoblotting of biofilm extracts for wild type (*lanes 1 and 4*), *tasA*/*sinR* (*lanes 2 and 5*), TasA-(170-369)EgA31 (*lane 3*) and TasA-(370-583)EgA31 (*lane 6*) strains detected with anti-TasA (*upper panel*) and anti-EgA31 (*lower panel*) antibodies. The* white arrowhead* indicates the position of the TasA-EgA31 antigenic peptides and the* red arrow* indicates the position of TasA. The protein molecular weights marker (kDa) is indicated

To simplify our expression system in biofilms, we engineered a non-domesticated *B. subtilis* NCBI 3610 strain by double deletion of *tasA* and *sinR* genes through replacement with a unique antibiotic gene resistance (*tasA/sinR::km*^*R*^, Table 1). As expected, no TasA was detected when tested a biofilm extract for the new *tasA/sinR* strain (Fig. 3a) by specific anti-TasA immunoblotting. Also, the biofilm colony architecture for the new *tasA/sinR* (Fig. 3c, first row) strain resulted similar to the above *tasA/sinR* with two antibiotic resistance genes (Fig. 2a, lower row). Thus, henceforth we will refer to the *tasA/sinR::km*^*R*^ strain as the preferred genetic background for heterologous expression.

**Table 1:** *Bacillus subtilis* strains used in this study

Strain	Genotype	Reference/source
168	Wild type domesticated	Kolter Lab., Harvard Medical School
NCIB3610	Wild type undomesticated	Kolter Lab., Harvard Medical School
*P*_*tapA*_-*yfp* (CA018)	*amyE::P*_*tapA*_-*yfp; Spc* ^*R*^	Vlamakis et al. [26]
*tasA* (CA017)	*tasA::Km* ^*R*^	Vlamakis et al. [26]
*sinR* (DS92)	*sinR::Spc* ^*R*^	Kearns et al. [46]
*tasA*/*sinR*	*tasA::Km* ^*R*^ *; sinR::Spc* ^*R*^	This study
TasA-mCherry(CA113)	*amyE::yqxM*-*sipW*-*tasA*-*mCherry; Cm* ^*R*^	This study
*tasA*/TasA-mCherry	*tasA::Km* ^*R*^ *; AmyE::yqxM*-*sipW*-*tasA*-*mCherry; Cm* ^*R*^	This study
*tasA*/*sinR*/TasA-mCherry	*tasA::Km* ^*R*^ *; sinR::Spc* ^*R*^ *; amyE::yqxM*-*sipW*-*tasA*-*mCherry; Cm* ^*R*^	This study
*tasA*/*sinR*	*tasA*-*sinR::Km* ^*R*^	This study
*tasA*/*sinR*/TasA-(102-207)EgTrp	*tasA*-*sinR::Km* ^*R*^ *; amyE::yqxM*-*sipW*-*tasA*-*(102*-*207)EgTrp; Spc* ^*R*^	This study
*tasA*/*sinR*/TasA-(102-278)EgTrp	*tasA*-*sinR::Km* ^*R*^ *; amyE::yqxM*-*sipW*-*tasA*-*(102*-*278)EgTrp; Spc* ^*R*^	This study
*tasA*/*sinR*/TasA-(170-369)EgA31	*tasA*-*sinR::Km* ^*R*^ *; amyE::yqxM*-*sipW*-*tasA*-*(170*-*369)EgA31; Spc* ^*R*^	This study
*tasA*/*sinR*/TasA-(370-583)EgA31	*tasA*-*sinR::Km* ^*R*^ *; amyE::yqxM*-*sipW*-*tasA*-*(370*-*583)EgA31; Spc* ^*R*^	This study

As a proof of principle, we tested a series of in-frame fusions of *Echinococcus granulosus* antigenic peptides to the C-terminus of TasA. As depicted in the schematic representation from Fig. 3b, we selected peptidic regions from tropomyosin (EgTrp) and paramyosin (EgA31) from *E. granulosus* that were previously described for their antigenic properties [35, 36]. For this purpose, we engineered four *B. subtilis* strains in a *tasA/sinR* background expressing TasA-(102-207)EgTrp, TasA-(102-278)EgTrp, TasA-(170-369)EgA31 or TasA-(370-583)EgA31. All these newly engineered strains were able to restore the deficient biofilm structure of the *tasA/sinR* strain in semi-solid (Fig. 3c, middle and lower rows) or liquid MSgg media (Fig. S1b). The TasA-EgTrp fusion proteins were detected in 72 h biofilm extracts by immunoblot, using specific anti-TasA and anti-EgTrp antibodies. As depicted in Fig. 3, it was possible to unmistakably distinguish the bands that corresponded to the predicted molecular weights of 40 kDa for TasA-(102-207)EgTrp (Fig. 3d, lanes 3 and 7) and 50 kDa for TasA-(102-278)EgTrp (Fig. 3d, lanes 4 and 8) fusion proteins. Similarly, extracts of biofilms expressing TasA-(170-369)EgA31 (Fig. 3e, lane 3) and TasA-(370-583)EgA31 (Fig. 3e, lane 6) showed a band of 43 and 46 kDa (Fig. 3e, upper panels), when using the anti-TasA antibody. We could detect only a weak signal for TasA-(170-369)EgA31, suggesting low expression levels. This effect was even more dramatic when using the anti-EgA31 antibody, where the fusion protein was undetectable under the conditions used in this assay. It is common to obtain small amounts of degradation when engineering and expressing fusion proteins. In this particular case, in fact, we were able to detect degradation from TasA-EgTrp and TasA-EgA31 fusion with anti-TasA antibodies but not with the specific anti-EgTrp and anti-EgA31, suggesting that the N-terminus of TasA is slightly unstable.

**Fig. 4:**
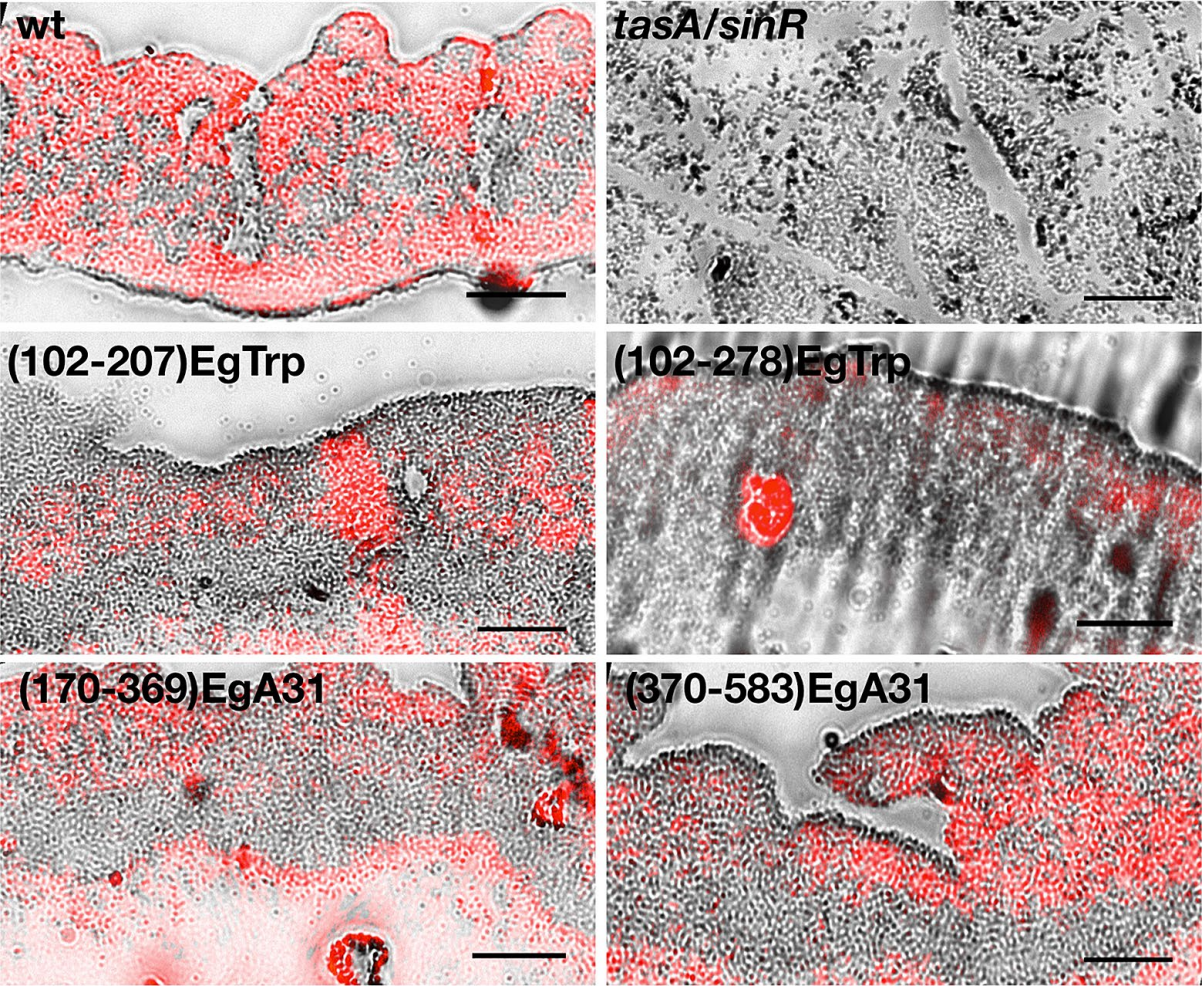
Detection of TasA fusion protein in biofilms by immunohistochemistry. Biofilms were grown for 72 h and then were formalin-fixed, paraffin-embedded and treated for immunohistochemistry. Images correspond to partial section of the colonies. TasA and TasA fused to *E. granulosus* antigenic peptides for EgTrp and EgA31 were detected using a specific anti-TasA antibody followed by a secondary antibody conjugated to Alexa-594 (*red*). Transmitted light images were overlaid with fluorescence images. The agar surface is at the bottom of the each image. *Scale bar* is 50 µm

We then examined the localization of the TasA fusion proteins within the biofilm by performing anti-TasA immunohistochemistry of 72 h biofilm sections. As expected (Fig. 4, upper row), TasA from wild type strain was distributed in the whole biofilm, while the *tasA/sinR* strain was negative for anti-TasA. The results obtained from biofilms expressing TasA fused to *E. granulosus* peptides (Fig. 4, middle and lower rows), exhibited the fusion protein from all the inspected sections in the biofilm matrix. Despite a relative uniform distribution of TasA was observed in the wild type strain, the TasA fused to *E. granulosus* peptides in a *tas/sinR* background showed an evident patchy distribution. This observation is consistent with the differences found in the reconstitution of the macro-structural architecture of the biofilm (Fig. 3) when comparing wild type strain to the ones expressing the TasA fusions. It is plausible that the tertiary structure from the peptides fused to TasA could influence the secretion of TasA to the extracellular matrix or alternatively, that the expression of TasA fused to peptides could alter the balance of the *B. subtilis* subpopulations within the biofilm.

**Fig. 5:**
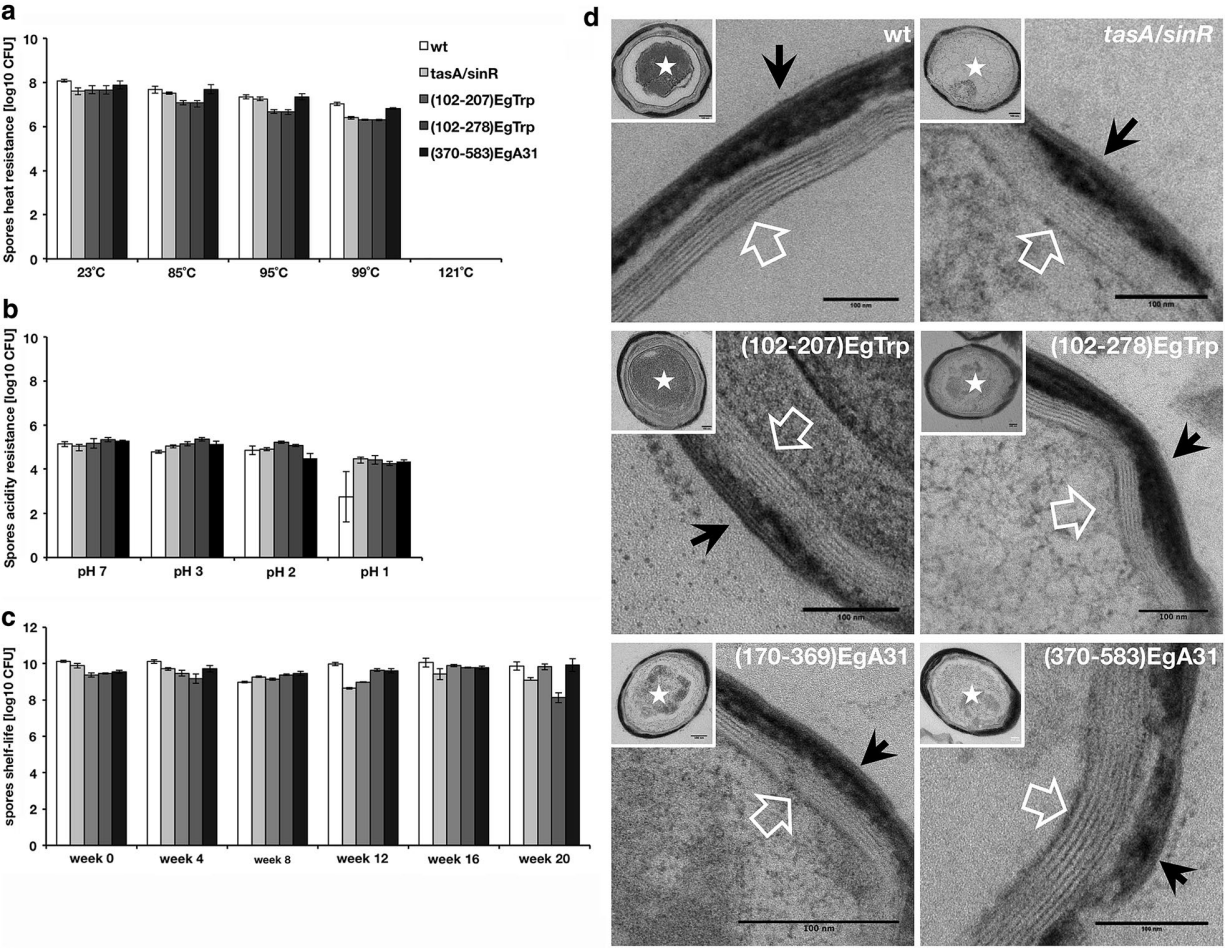
Wild type and recombinant spores display equal performances. Spores were tested under different conditions as: **a** heat resistance, **b** acidic medium and **c** shelf-life storage. Data represent the mean ± SEM from three independent experiments (t-test, not significant, p value >0.05). *Error bars* indicate SEM. **d** Transmission electron microscopy of wild type, *tasA*/*sinR,* TasA-(102-207)Egtrp, TasA-(102-278)EgTrp, TasA-(170-369)EgA31 and TasA-(370-583)EgA31 spore strains. Spores were frozen with liquid nitrogen, fixed with glutaraldehyde, counterstained and photographed. *Black arrowhead,* spore coats*; white arrowhead,* spore cortex peptidoglycan*; star,* spore protoplast. *Scale bars* are 50 and 100 nm, as indicated

We next investigated whether the recombinant spores carrying the heterologous *tapA*-*sipW*-*tasA*-*E. granulosus peptide* operon has the same biophysical features as the wild type spores, testing for their capacity to endure in harsh conditions as high temperature, acidic environment and also, shelf-life viability. Upon all these conditions (Fig. 5a–c) the recombinant spores were not significantly different from the wild type. Moreover, when examining the ultrastructure of the spores by high-definition electron microscopy (Fig. 5d), the spore coats and the spore cortex peptidoglycan showed identical structures in all the inspected spores. Taken together, these results provide strong evidence showing that the recombinant spores have indistinguishable biophysical and morphological properties than the wild type spores, suggesting similar resistance to harsh environments, protection of the genetic material and germination when conditions are appropriate.

## Conclusions

In this work, we provide strong evidence showing that TasA, an important matrix protein for biofilm formation in *B. subtilis,* can be used for the display of proteins of interest within the biofilm. Such expression was demonstrated using proteins from diverse origins, as are the red fluorescent protein mCherry and the antigenic peptides from *E. granulosus* parasite, Egtrp and EgA31. By deleting the *tasA* and *sinR* genes in a non-domesticated *B. subtilis* NCBI3610 strain, we were able to enhance the expression of the fusion proteins. We show that our methodology can be used to effectively express the heterologous proteins within the biofilm. This was demonstrated by the reestablishment of macrostructures in an architecture-deficient biofilm background, the detection of the fusion proteins in biofilm extracts by immunoblot and their localization in biofilm histological cuts. Finally, we portrayed that the preserved characteristic of the recombinant spores, due their capacity to protect the integrated recombinant *tapA*-*sipW*-*tasA*-*gene peptide* operon, could provide an excellent tool to facilitate the transport and desired location for a biofilm to display a heterologous protein of interest.

## Methods

### *B. subtilis* strains, media and culture conditions

A list with the *B. subtilis* strains used in this study is showed in Table 1. For routine growth and spore quantification, cells were propagated on Luria–Bertani (LB) medium. For biofilm assays, cells were scraped from overnight growth on LB-agar plates, resuspended in LB liquid medium to an OD_600_ of 1, and then 2 μL of this suspension were spotted on MSgg solid medium or in 2.5 mL of MSgg medium in 22 mm^2^-well plates [23]. Biofilms were incubated at 30 °C. The final concentration of antibiotics used for the *B. subtilis* strains were the following: spectinomycin (Spc) (100 µg/mL), kanamycin (Km) (10 µg/mL) and chloramphenicol (Cm) (5 µg/mL).

### Antibodies and reagents

Rabbit polyclonal anti-TasA was a gift from Dr. R. Losick (Harvard University, Cambridge, USA). Mouse polyclonal anti-EgTrp and mouse polyclonal anti-EgA31 were a gift from Dr. M-F. Pétavy (Université Claude Bernard Lyon 1, Lyon, France). Rabbit anti-mouse F (ab’)2 fragments Alexa 594 was obtained from Molecular Probes, Invitrogen, USA. Goat polyclonal anti-mouse IgG (Fab)’-peroxidase and goat polyclonal anti-rabbit Ig-peroxidase were obtained from Sigma-Aldrich.

### Plasmid constructions

The plasmid pDG-TasA-mCherry was obtained by digestion of pBS-TapAop-mCherry with *Bam*HI restriction enzyme to obtain TapAop-mCherry and ligated in the corresponding sites of pDG1662 [37]. pBS-TapAop-mCherry was obtained by PCR amplification of mCherry from pRSET-mCherry [38] using specific primers to insert XbaI and *Bam*HI/*Sac*I restriction sites respectively and, subsequently, ligated in pBS-TapAop between *Xba*I and *Sac*I restriction sites. The plasmid pBS-TapAop was obtained by PCR amplification of the *tapA*-*sipW*-*tasA* operon from genomic DNA of *B. subtilis* NCBI3610 using specific primers to insert *Bam*HI and *Xba*I restriction sites. The fragment was ligated XbaI and BamHI in pBluescript II KS(+)(Stratagene).

The plasmids pDG-TasA-(102–207)EgTrp, pDG-TasA-(102–278)EgTrp, pDG-TasA-(170–369)EgA31 and pDG-TasA-(370–583)EgA31 were obtained by digestion of the plasmids pBS-TapAop-(102–207)EgTrp, pBS-TapAop-(102–278)EgTrp, pBS-TapAop-(170–369)EgA31 and pBS-TapAop-(370–583) with *Xho*I and *Bam*HI restriction enzymes to obtain the fragments TapAop-(102–207)EgTrp, TapAop-(102–278)EgTrp, TapAop-(170–369)EgA31 and TapAop-(370–583)EgA31, respectively. The fragments were then ligated into pDG1730 [37] between *Xho*I and *Bam*HI restriction sites. The plasmids pBS-TapAop-(102–207)EgTrp, pBS-TapAop-(102–278)EgTrp, pBS-TapAop-(170–369)EgA31 and pBS-TapAop-(370–583) were obtained by PCR amplification of EgTrpA and EgA31 fragments from the constructs pQIA-EgTrp and pQIA-EgA31 (gently provided by Dr. Adriana Esteves, Universidad de la República, Montevideo, Uruguay) [39, 40] using specific primers containing flanking *Not*I, *Bam*HI and *Sma*l restriction sites (Table 2), followed by ligation between NotI and SmaI in pBS-TasAop(SSSN). The plasmid pBS-TasAop(SSSN) was obtained by PCR amplification of *tapA*-*sipW*-*tasA* operon fragment from pBS-TapAop-mCherry using specific primers containing XhoI and NotI restricton sites, followed by ligation between XhoI and NotI in pBS(SSSN). The plasmid pBS-MCS(SSSN) was obtained by ligation of the annealed oligonucleotides (5′-ATGCCTCGAGGGATCCTCAGAGTTAAATGGTATTGCT-3′ and 5′-GCATGCGGC CGCATTTTTATCCTCGCTATGCGC-3′) between *Sal*I and *Not*I restriction sites in pBluescript-KSII(+) (Stratagene). All oligonucleotides were obtained from Microsynth AG, Switzerland and described in Table 2.

**Table 2:** Primers used for plasmid construction

Amplified segment	Oligonucleotide sequences
*tasA*-*sipW*-*tasA* operon(1)	Fwd: 5′- ATGCGGATCCTCAGAGTTAAATGGTATTGCT-3′ Rev: 5′-GCATTCTAGAATTTTTATCCTCGCTATGCGC
*tasA*-*sipW*-*tasA* operon(2)	Fwd.: 5′-ATGCCTCGAGGGATCCTCAGAGTTAAATGGTATTGCT-3′ Rev.: 5′-GCATGCGGCCGCATTTTTATCCTCGCTATGCGC-3′
mCherry	Fwd.: 5′-GATCTCTAGA **ATG**GTGAGCAAGGGCGAGGAG-3′ Rev.: 5′-GATCGAGCTCGGATCC **TTA**CTTGTACAGCTCGTCCAT-3′
(102-207)EgTrp	Fwd.: 5′ ATGCGCGGCCGCCGAAACATCTACTAAGCTTGAC-3′ Rev.: 5′ GATCCCCCGGGGGATCC **TTA**CTCTTGCTCGGAGACTTCGAG-3′
(102-278)EgTrp	Fwd.: 5′ ATGCGCGGCCGCCGAAACATCTACTAAGCTTGAC-3′ Rev.: 5′ GATCCCCGGGGGATCC **TCA**GAAGGAAGTGAGCTCCGC-3′
(170-369)EgA31	Fwd.: 5′ ATGCGCGGCCGCCGCAGCT5GAAAAACAAGCCATG-3′ Rev.: 5′ GATCCCCGGGGGATCC **TCA**CCTTGTTTCAAGCATTTCAAT-3′
(370-583)EgA31	Fwd.: 5′ ATGCGCGGCCGCCGCTGAGACTAAAGAAATTAAT-3′ Rev.: 5′ GATCCCCGGGGGATCC **TCA**ATCTCTTTCGAGCTGTTTGAT-3′

The integrated restriction sites are underlined

The start and stop codons are label in bold

The long-flanking homology PCR (LFH–PCR) technique was used for creating the deletion mutant *tasA*-*sinR*::*Km*^*R*^ by means of gene replacement, as described by Vlamakis et al. [26]. For this purpose, a joining PCR to create *tasA*-*Km*^*R*^-*sinR* was prepared from genomic DNA from *B. subtilis* NCIB3610, amplifying *tasA* with FtasAUP (5′-ACAATAAGTCATGGCCGGA-3′) and RtasADO (5′-CCTATCACCTCAAATGG TTCGCTGGTTCGCTGGTTTAATACGCTGGCCAA-3′) and *sinR* with FsinRUP (5′-CGAGCGCCTACGAGGAATTTGTATCGGCTCCCCTTTTATTGAATG-3′) and RsinRDO (5′-TATGCCGGCTATATGCTT-3′). The *Km*^*R*^ gene was obtained by amplification of genomic DNA from *B. subtilistasA* (CA017) strain [26] using the primers FKmrUP (5′-CAGCGAACCATTTGAGGTGATAGG-3′) and RKmrDo (5′-CGATACAAATTCCTCGTAGGCGCTCGG-3′). A *B. subtilis* 168 *tasA/sinR::Km*^*R*^ was used as donor strain for transferring the mutant allele into the *B. subtilis* strain NCIB3610 by means of SPP1-mediated generalized transduction [41].

### Transformation of *B. subtilis*

*Bacillus subtilis* strain 168 was transformed routinely as described by Cutting and Vander Horn [42]. Briefly, bacteria were inoculated in 10 mL of LB medium and grown overnight at 37 °C with agitation. After centrifugation, the bacterial pellet was resuspended in transformation medium (6 mM K_2_HPO_4_, 4 mM KH_2_PO4, 10 mM d-glucose, 100 mg/L casamino acids, 1.5 mM L-glutamate, 300 mM sodium citrate, 4 mM ferric ammonium citrate, 0.3 mM MgSO_4_, 25 µM tryptophan and 25 µM phenylalanine) and let grow to early stationary phase (OD_600 nm_ of 1.25). Then, 1 mL of bacteria were mixed with 5 µg of plasmid DNA, and the tubes were rolled for 40 min at 37 °C. The cells were plated on LB agar medium supplemented with antibiotics. The transformants were selected with the appropriate antibiotics for a double crossover recombination at the *amyE* locus [37]. The diverse *tapA*-*sipW*-*tasA*-*fusion gene* operons were then transferred to NCIB3610 by SPP1-mediated generalized transduction [41]. The positive clones were identified by direct PCR of the selected colonies using the following specific primers pDG5′-F (5′-ATAATTTTAAATGTAAGCGTT-3′) and pTasAop-R (5′-CTGTAAAAGAAGCAAAAAAAAA-3′).

### Quantification of mCherry fluorescence in biofilms

The biofilm fluorescence (photon/sec) and area (mm^2^) were measured at 24, 48, and 72 h post-inoculation, using NightOWL LB 983 (Berthold Technologies). Statistical analysis was performed with the aid of Microsoft^®^ Excel^®^ for Mac 2011, version 14. 6.2.

### Preparation of *B. subtilis* spores

*Bacillus subtilis* spores were prepared as described by Nicholson and Setlow [34]. Briefly, a colony of *B. subtilis* was inoculated in 3 mL LB media supplemented with antibiotics and incubated at 37 °C for 18 h in a horizontal shaker at 180 rpm. The inoculum was expanded in 500 mL LB media in a 2 L Erlenmeyer flask and then grown for 12 h at 37 °C in a shaker at 180 rpm. Afterwards, 50 mL of the culture were diluted in 500 mL of DSM (8 g/L bacto nutrient broth, 13.4 mM KCl, 2 mM MgSO_4_, 1 mM Ca(NO_3_)_2_, 10 µM MnCl_2_ and 1 µM FeSO_4_) contained in a 2 L Erlenmeyer Flask and incubated for 72 h at 37 °C in a shaker at 180 rpm. The spores were harvested and centrifugated at 14,000×*g*, for 10 min and 4 °C, and the pellet heated for 30 min at 80 °C. The pellet was subsequently washed with ten volumes of the following solutions: a) 1 M KCl and 0.5 M NaCl; b) 50 mM TrisCl pH 7.2 and lysozyme [50 µg/mL], incubated for 60 min at 37 °C; c) 1 M NaCl and d) twice with ten volumes of deionized water. After each wash, the spores were centrifugated at 14,000×*g*, for 10 min and 4 °C. Finally, the pellet was resuspended in deionized water and stored at −80 °C for further use. The spore quantification was determined as described by Vlamakis et al. [26].

### Quantification of spores in biofilm

The ability of recombinant *B. subtilis* to sporulate in a biofilm was determined essentially as described by Vlamakis et al. [26]. Briefly, *B. subtilis* cultured in LB media were diluted to OD_600 nm_ of 1 and 10 µL of the suspension were inoculated, in duplicate, over 2.5 mL of MSgg media in 12-well culture plates. The plates were incubated at room temperature with no agitation. Samples of cells were taken after 48 h and subjected to mild sonication conditions (10 s at 14 kHz) to obtain intact single cells. The optical density of each preparation was normalized to OD_600 nm_ of 1 after sonication. To kill vegetative cells, the normalized preparations were incubated for 20 min at 80 °C. To determine viable spore counts, serial dilutions were plated from the normalized preparation after the 80 °C incubation.

### Immunoblotting

Biofilms in MSgg agar were harvested at 72 h in 500 µL of deionized water followed by homogenization by sonication. Samples were normalized to OD_600 n_ of 1. Then, 20 µL of the normalized sample was mixed with 5 µL sample buffer 4× (8 % SDS, 40 % Glycerol, 200 mM Tris pH 6.8, 4 % 2-mercaptoethanol, 0.4 % Bromophenol blue) and heated for 5 min at 95 °C. Samples were further processed as described by Eichwald et al. [43].

### Biofilm imaging

Whole colonies were photographed at low magnification (0.8×) using a Wild Heerbrugg M5A stereomicroscope equipped with a 1.25 × S objective lens, a Leica DFC420 digital camera, and a computer interface. Data were captured using LAS V4 (Leica). Pictures were processed using ImageJ, version: 2.0.0-rc-44/1.50e software (Creative Commons license).

### Stability of *B. subtilis* recombinant spores

For each experiment, *B. subtilis* spores were diluted in deionized water and identical aliquots were prepared in screw lid tubes. For shelf life stability, spores were stored for the indicated times in dark at room temperature. For temperatures resistance, the samples were heated for 30 min at the indicated temperatures. For resistance to acidic environment, the spores were centrifugated for 5 min at 10,000×*g*, the pellet resuspended in 500 µL of a solution at different pHs and incubated for 2 h at 37 °C. The number of spores was determined as CFU/mL as described by Vlamakis et al. [26]. Experiments were performed in triplicate.

### Electron microscopy

A 3 µL aliquot of freshly prepared spores was placed on small pieces (2 × 3 mm) of 4 % trypticase soy agar mounted on cigarette paper and slammed on a highly polished copper block cooled by liquid nitrogen in a freezing device (KF 80, Reichert-Jung, Austria). Then, the samples were transferred to a freeze substitution unit (FS 7500, Boeckler Instruments, Tucson, AZ, USA) precooled at −88 °C for substitution with acetone and subsequently, fixed with 0.25 % glutaraldehyde and 0.5 % osmium tetroxide rising the temperatures gradually to +2 °C [44], keeping the temperature at −88 °C for up to 2 days. Then, the samples were embedded in epon followed by polymerization at 60 °C for 2.5 days. Ultrathin Section (70–80 nm) were cut and stained with uranyl-acetate and lead-citrate before analysis in a transmission electron microscope (CM12, Philips, Eindhoven, The Netherlands) equipped with a CCD camera (Ultrascan 1000, Gatan, Pleasanton, CA, USA) at an acceleration voltage of 100 kV. Data were analyzed with ImageJ, version: 2.0.0-rc-44/1.50e software (Creative Commons license).

### Immmunohistochemistry of biofilms

A 72 h post-inoculated biofilm, grown in MSgg medium fortified with 1.5 % agar, was harvested, formalin-fixed and paraffin-embedded [45]. Semi-thin Section (2–3 µm) were placed in gelatin pre-coated glass-slides, deparafinizzed by immersion in xylene and rehydrated by immersion in decreasing ethanol gradient (95, 70 and 50 %). The slides were immersed in phosphate buffer saline solution (PBS)(10 mM H_3_PO_4_, 137 mM NaCl, 2.7 mM KCl) followed by blocking in a 5 % BSA in PBS in a humid chamber. The primary antibody (rabbit polyclonal anti-tasA antibody, dilution 1:1000) was incubated for 90 min at room temperature in a humid chamber, washed twice for 3 min in 0.025 % Triton X-100 in PBS and then followed by two washes of 3 min in PBS. The secondary antibody (anti-rabbit conjugated to Alexa 488, dilution 1:500) was incubated for 60 min at room temperature in a humid chamber. Then, slides were covered with a glass coverslip using Prolong Diamond mounting media (Molecular Probes). Images were acquired using a fluorescent microscope (Leica DMI 6000B) equipped with an HCX/PL/Fluotar 20X objective. All images were acquired with the same exposure time and processed using ImageJ, version: 2.0.0-rc-44/1.50e software (Creative Commons license).

## Additional files

10.1186/s12934-016-0532-5 (**a**) Top view of 48 h pellicle formation of *B. subtilis* strains: wild type (3610) (left), *tasA/sinR* (middle) and *tasA/sinR*/TasA-mCherry (right), incubated in MSgg medium at 30°C without agitation. (**b**) Top view of 48h pellicle formation of *B. subtilis* strains: wild type (3610), *tasA*/*sinR*, TasA-(102-207)EgTrp, TasA-(102-278)EgTrp, TasA-(170-369)EgA31 and TasA-(370-583)EgA31, incubated in MSgg medium at 30°C without agitation. (**c**) Immunoblotting of 48h pellicle extracts of *B. subtilis*: wild type (lane 1), *tasA* (lane 2), *tasA/sinR* (lane 3), TasA-mCherry (lane 4), *tasA*/TasA-mCherry (lane 5) and *tasA/sinR*/TasA-mCherry (lane 6) detected with anti-TasA (upper panel) and anti-dsRed2 (lower panel). Red arrows indicate the positions for TasA-mCherry and TasA. The red bracket indicates an anti-TasA reactive band of a lower molecular weight than TasA, presumably TasA degradation. The protein molecular weights marker is indicated.
10.1186/s12934-016-0532-5 Transmission electron microscopy of *B. subtilis* spores wild type (3610), *tasA*, *sinR* and *tasA*/*sinR* spore strains. Spores were frozen in liquid nitrogen, fixed with glutaraldehyde, counterstained and photographed. Black arrowhead, spore coat; white arrowhead, spore cortex peptidoglycan; star, spore protoplast. Scale bar is 0.2 µm.
10.1186/s12934-016-0532-5 Viable spore counts comparing TasA-mCherry, *tasA*/TasA-mCherry and *tasA*/*sinR*/TasA-mCherry to wild type percent of spores in 72h biofilms or liquid media.

## Abbreviations

PBS: phosphate buffer saline
BSA: bovine serum albumin
CFU: colony forming unit
