# Supplementary figures and images for: Heterologous expression of antigenic peptides in Bacillus subtilis biofilms
Source: Microb Cell Fact. 2016 Aug 11;15:137. doi: 10.1186/s12934-016-0532-5 (PMC4982213; doi:10.1186/s12934-016-0532-5)

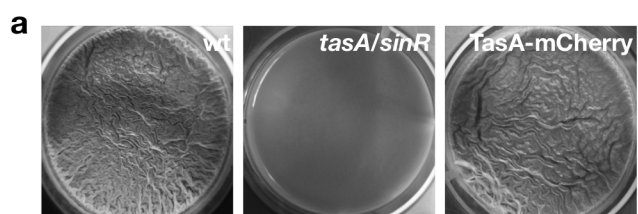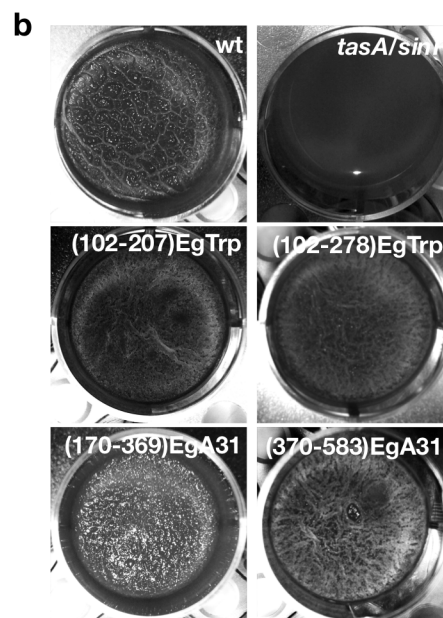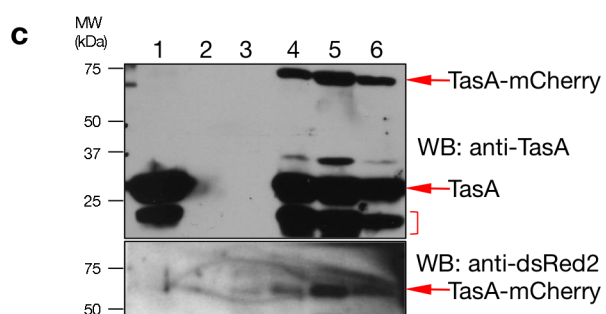

Supplement: Supplementary file 1 — 10.1186/s12934-016-0532-5 (a) Top view of 48 h pellicle formation of B. subtilis strains: wild type (3610) (left), tasA/sinR (middle) and tasA/sinR/TasA-mCherry (right), incubated in MSgg medium at 30°C without agitation. (b) Top view of 48h pellicle formation of B. subtilis strains: wild type (3610), tasA/sinR, TasA-(102-207)EgTrp, TasA-(102-278)EgTrp, TasA-(170-369)EgA31 and TasA-(370-583)EgA31, incubated in MSgg medium at 30°C without agitation. (c) Immunoblotting of 48h pellicle extracts of B. subtilis: wild type (lane 1), tasA (lane 2), tasA/sinR (lane 3), TasA-mCherry (lane 4), tasA/TasA-mCherry (lane 5) and tasA/sinR/TasA-mCherry (lane 6) detected with anti-TasA (upper panel) and anti-dsRed2 (lower panel). Red arrows indicate the positions for TasA-mCherry and TasA. The red bracket indicates an anti-TasA reactive band of a lower molecular weight than TasA, presumably TasA degradation. The protein molecular weights marker is indicated. [file 12934_2016_532_MOESM1_ESM.pdf]

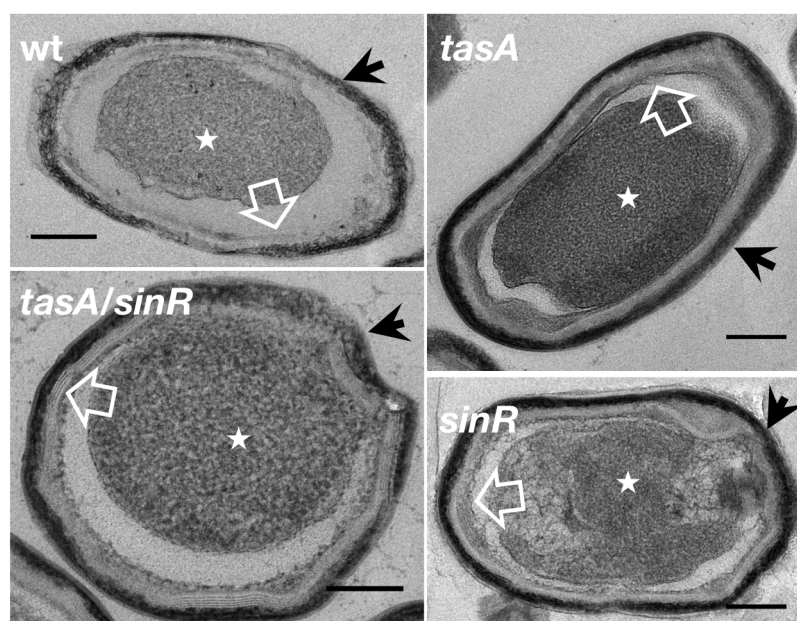

Supplement: Supplementary file 2 — 10.1186/s12934-016-0532-5 Transmission electron microscopy of B. subtilis spores wild type (3610), tasA, sinR and tasA/sinR spore strains. Spores were frozen in liquid nitrogen, fixed with glutaraldehyde, counterstained and photographed. Black arrowhead, spore coat; white arrowhead, spore cortex peptidoglycan; star, spore protoplast. Scale bar is 0.2 µm. [file 12934_2016_532_MOESM2_ESM.pdf]

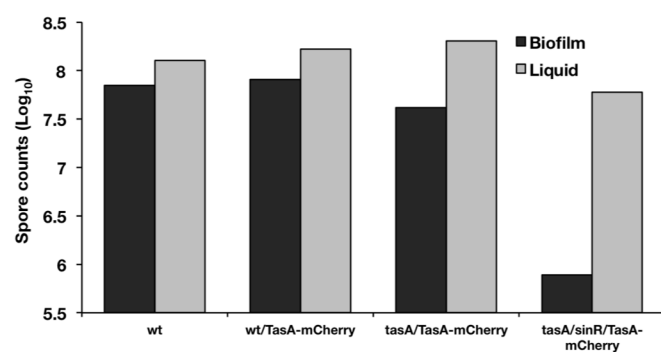

Supplement: Supplementary file 3 — 10.1186/s12934-016-0532-5 Viable spore counts comparing TasA-mCherry, tasA/TasA-mCherry and tasA/sinR/TasA-mCherry to wild type percent of spores in 72h biofilms or liquid media. [file 12934_2016_532_MOESM3_ESM.pdf]
